# CRISPR/Cas-Assisted Nanoneedle Sensor for Adenosine Triphosphate Detection in Living Cells

**DOI:** 10.1021/acsami.3c07918

**Published:** 2023-09-28

**Authors:** Hongki Kim, Chenlei Gu, Salman Ahmad Mustfa, Davide Alessandro Martella, Cong Wang, Yikai Wang, Ciro Chiappini

**Affiliations:** † Centre for Craniofacial and Regenerative Biology, King’s College London, London SE1 9RT, U.K.; ‡ Department of Chemistry, Kongju National University, Gongju 32588, Republic of Korea; § London Centre for Nanotechnology, King’s College London, London SE1 9RT, U.K.

**Keywords:** sensor, biosensor, nanomedicine, CRISPR/Cas, nanoneedles, ATP sensing, porous silicon

## Abstract

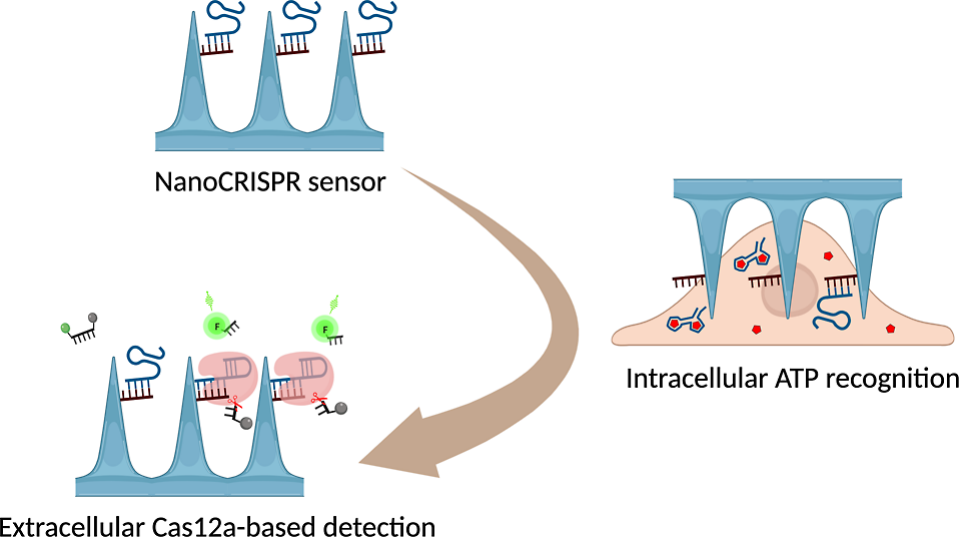

The clustered regularly interspaced short palindromic repeats (CRISPR)-associated protein (Cas) (CRISPR/Cas) systems have recently emerged as powerful molecular biosensing tools based on their collateral cleavage activity due to their simplicity, sensitivity, specificity, and broad applicability. However, the direct application of the collateral cleavage activity for in situ intracellular detection is still challenging. Here, we debut a CRISPR/Cas-assisted nanoneedle sensor (nanoCRISPR) for intracellular adenosine triphosphate (ATP), which avoids the challenges associated with intracellular collateral cleavage by introducing a two-step process of intracellular target recognition, followed by extracellular transduction and detection. ATP recognition occurs by first presenting in the cell cytosol an aptamer-locked Cas12a activator conjugated to nanoneedles; the recognition event unlocks the activator immobilized on the nanoneedles. The nanoneedles are then removed from the cells and exposed to the Cas12a/crRNA complex, where the activator triggers the cleavage of an ssDNA fluorophore-quencher pair, generating a detectable fluorescence signal. NanoCRISPR has an ATP detection limit of 246 nM and a dynamic range from 1.56 to 50 μM. Importantly, nanoCRISPR can detect intracellular ATP in 30 min in live cells without impacting cell viability. We anticipate that the nanoCRISPR approach will contribute to broadening the biomedical applications of CRISPR/Cas sensors for the detection of diverse intracellular molecules in living systems.

## Introduction

Recently, the clustered regularly interspaced short palindromic repeats (CRISPR)-associated nuclease (Cas) (CRISPR/Cas) system, originally identified in bacteria and archaea, has gained considerable attention in molecular diagnostics due to its superb specificity of molecular target recognition, fast turnaround time, convenient isothermal reaction, and signal amplification capabilities.^1–3^ Several CRISPR-based sensing approaches such as DETECTR (DNA endonuclease-targeted CRISPR trans reporter), SHERLOCK (specific high-sensitivity enzymatic reporter unlocking), HOLMES (1 h low-cost multipurpose highly efficient system), and others, have been developed with high sensitivity and specificity.^4–9^ For example, DETECTR showed attomolar sensitivity; HOLMES achieved a limit of detection (LOD) around 10 aM, and SHERLOCKv2 enabled the multiplexed sensing of target detection at zeptomolar sensitivity.^7–9^ Among diverse Cas proteins, Cas12a (a class of type V nuclease) shows a unique collateral cleavage activity that can nonspecifically cleave single-stranded DNA substrates (termed as *trans*-cleavage) upon recognition of the correct target DNA by crRNA.^7,9^ By adopting single-stranded DNA labeled with a fluorophore and quencher pair, the collateral cleavage activity can generate enormously amplified signals in response to a target analyte, including not only nucleic acids but also proteins, transcription factors, and small molecules.^10–12^ In particular, Cas12a-based sensors can rapidly and sensitively detect adenosine triphosphate (ATP) levels.^13–17^ Yet Cas12a collateral cleavage is not suited to in situ intracellular detection as it requires the codelivery of multiple components (the Cas12a RNP, the crRNA, and the DNA substrates) and risks indiscriminate off-target cleavage of nucleic acids essential to cell function.^18,19^ Such a limitation prevents the use of this sensitive and specific sensing approach to monitor live cells.

Nanomaterial-based approaches are well suited to enable intracellular sensing.^1^ Numerous strategies involving nanoparticles, nanorods, and nanowires have been extensively explored.^20,21^ In particular, nanoneedles, vertically aligned arrays of high aspect ratio nanostructures, have been widely used to access the intracellular space with minimal disruption of cell functions.^22,23^ Nanoneedles can efficiently deliver nucleic acids within cells, with key applications for advanced therapies^24,25^ including topical genetic engineering^26,27^ and CAR-T cell manufacturing.^28^ Furthermore, such nonperturbing intracellular access enables efficient sensing of intracellular biomolecules.^23,26,29–32^ These unique functionalities of nanoneedles are attracting significant interest in the development of biomedical products.^33^ In particular, porous silicon nanoneedles have a high surface area to volume ratio which provides abundant binding sites for target capture/interaction, with the potential to enhance sensing performance.^26,29–31^ They are bioresorbable, making them suitable for in vivo diagnostics with minimal concerns for patient health. Porous silicon nanoneedles can successfully detect the intracellular activity of tumor biomarker enzymes in living cells and clinical samples using fluorescent-based approaches. While enzymatic biomarkers can be detected intracellularly, relying on their intrinsic signal amplification capacity, detecting nonenzymatic biomarkers is more challenging. Nonetheless, nanoneedles decorated with suitable capture probes can effectively “fish” target biomolecules from living cells, and do so repeatedly for longitudinal analysis of cell state.^34–37^ Fished molecules can be detected using ex situ amplification strategies.^38^ Such capability provides a unique avenue to combine the specificity and sensitivity of Cas12a-based sensing with unprecedented access to the intracellular space provided by nanoneedles for the detection of nonenzymatic intracellular targets.

In particular, ATP is the primary energy carrier molecule for living organisms, playing a vital role in biochemical reactions and cellular metabolic processes.^39^ The intracellular levels of ATP are tightly regulated to ensure cell health and direct key biological processes, including cell division, self-renewal, and differentiation.^40,41^ ATP assays are a cornerstone of molecular biology for monitoring cell viability, proliferation, and cytotoxic events, yet to date, ATP quantification is a destructive process requiring cell lysis. Monitoring ATP dysregulation can provide diagnostic information for cardiovascular, neurodegenerative, and mitochondrial diseases, hypoxia, hypoglycemia, ischemia, and the progression, invasiveness, and drug resistance of malignant tumors.^40,42–48^ Therefore, accurate detection and quantification of ATP are highly important to understand biological systems and improve diagnosis and treatment. Luciferase-based approaches for ATP detection are a workhorse in molecular biology, with good sensitivity for quantification from cell lysates.^49^ Other methods for ATP analysis include electrophoresis, isotope tracing, and high-performance liquid chromatography.^50–52^ Although these methods have been proven to be effective for ATP detection in vitro, they are time-consuming, require tedious sample preparations, and cannot be used in living cell. FRET-based approaches can monitor ATP levels with single cell resolution in live system but use genetically encoded reporters which require complex genetic engineering and cannot be deployed in a diagnostic setting.^53^ Thanks to their strong potential for signal amplification, Cas12-based approaches are emerging for the intracellular detection of biomolecules, including ATP.^54,55^

In this study, we detect intracellular ATP levels in a live cell culture using a nanoneedle-based approach that relies on Cas12a amplification. We functionalized the surface of porous silicon nanoneedles with a Cas12 activator locked by an ATP aptamer. The nanoneedles are interfaced with cells displaying the sensing element in the cytosol; upon intracellular ATP recognition, the configurational changes of the aptamer release the activator. The retrieved nanoneedles are incubated with the Cas12 detection system, which provides an assessment of the relative intracellular ATP concentration in the cell culture.

## Results

### NanoCRISPR: The CRISPR/Cas-Assisted Nanoneedle Sensor

**Figure 1:**
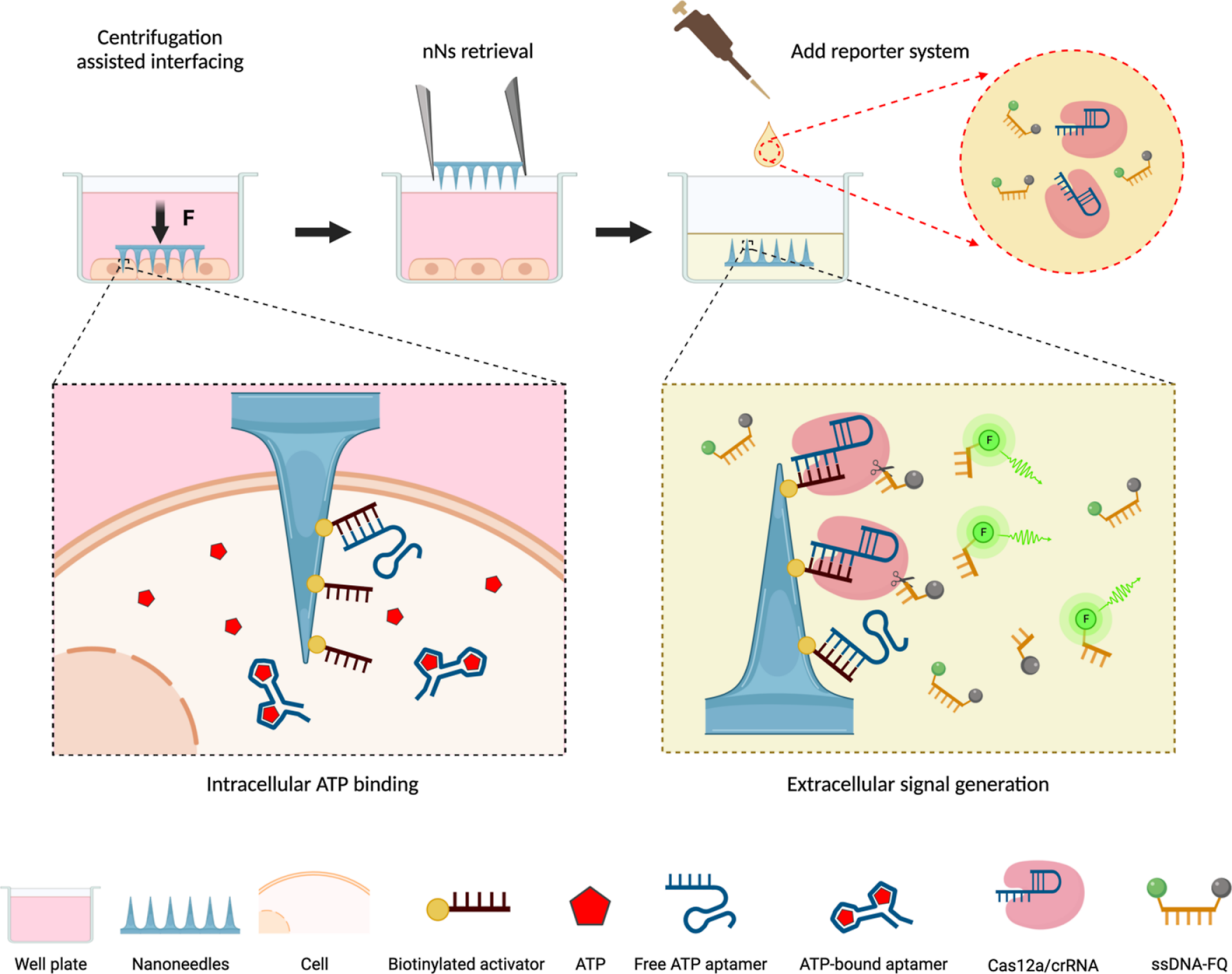
Schematic representation of the CRISPR/Cas-assisted nanoneedle sensor for the intracellular detection of ATP. The locked activator-modified nanoneedles are placed in the cell culture well with the nanoneedles facing toward cells (nanoneedles on top interfacing), and the whole setup is centrifuged. The centrifugation interfaces the nanoneedles with the cells, presenting the aptamers in the cytosol where they bind to intracellular ATP. When bound to ATP, the aptamer is released and unlocks the activator. After centrifugation, the nanoneedles are retrieved and incubated with the reporter system containing a Cas12a/crRNA complex and a ssDNA F-Q. The exposed activator triggers the cleavage of ssDNA-FQ by Cas12a/crRNA, yielding a detectable fluorescent signal.

NanoCRISPR is a CRISPR/Cas-assisted nanoneedle sensor for ATP detection, which operates in two steps. It first exploits the intracellular access provided by nanoneedles to expose an aptamer-locked CRISPR/Cas12 activator to cytosolic ATP, and then it generates an amplified signal by exposing the unlocked activator to CRISPR/Cas12 outside the cell (Figure 1). The sensor was assembled using conical porous silicon nanoneedles with 2.4 μm length, 150 nm tip diameter, 2 μm spacing, and 40% porosity. The dimensions of the nanoneedles were chosen for their efficiency accessing the intracellular space with minimal perturbation, as previously determined by our analysis of the nanoneedle biointerface.^26,29,30^ The surface of the nanoneedles was oxidized by an O_2_ plasma and then functionalized with 3-aminopropyltriethoxysilane (APTES) (Figure S1).

Biotin was conjugated to the amine-functionalized nanoneedles, followed by the addition of polystreptavidin. The locked activator composed of an ATP aptamer strand locking the biotin-labeled activator strand was immobilized onto nanoneedles through the interaction with the polystreptavidin. For intracellular ATP detection, the nanoneedles functionalized with the locked activator were centrifuged over HEK 293 adherent cells in culture with the needles facing toward cells. Assisted interfacing by centrifugation can increase membrane permeability, promoting access to the cell without additional perturbation.^23,56–59^ Following centrifugation, the nanoneedles were retrieved and exposed to a preassembled Cas12a/crRNA complex alongside single-stranded DNA labeled with a fluorophore (FAM) and a quencher (IABkFQ) (named ssDNA-FQ). During cell interfacing, the aptamer bound to the ATP present in the cell, undergoing a conformational change that led to its release and exposed the activator. The exposed activator remained on the nanoneedles and was specifically recognized when exposed to Cas12a/crRNA, thereby activating the cleavage of ssDNA-FQ, yielding a fluorescent signal correlated with the amount of intracellular ATP, which was measured in a plate reader. With this approach, the synergistic combination of the nonperturbing intracellular access granted by nanoneedle array and the amplifying capacity of the CRISPR system could allow for simple and rapid detection of trace ATP in living cells.

### nanoCRISPR Assembly

**Figure 2:**
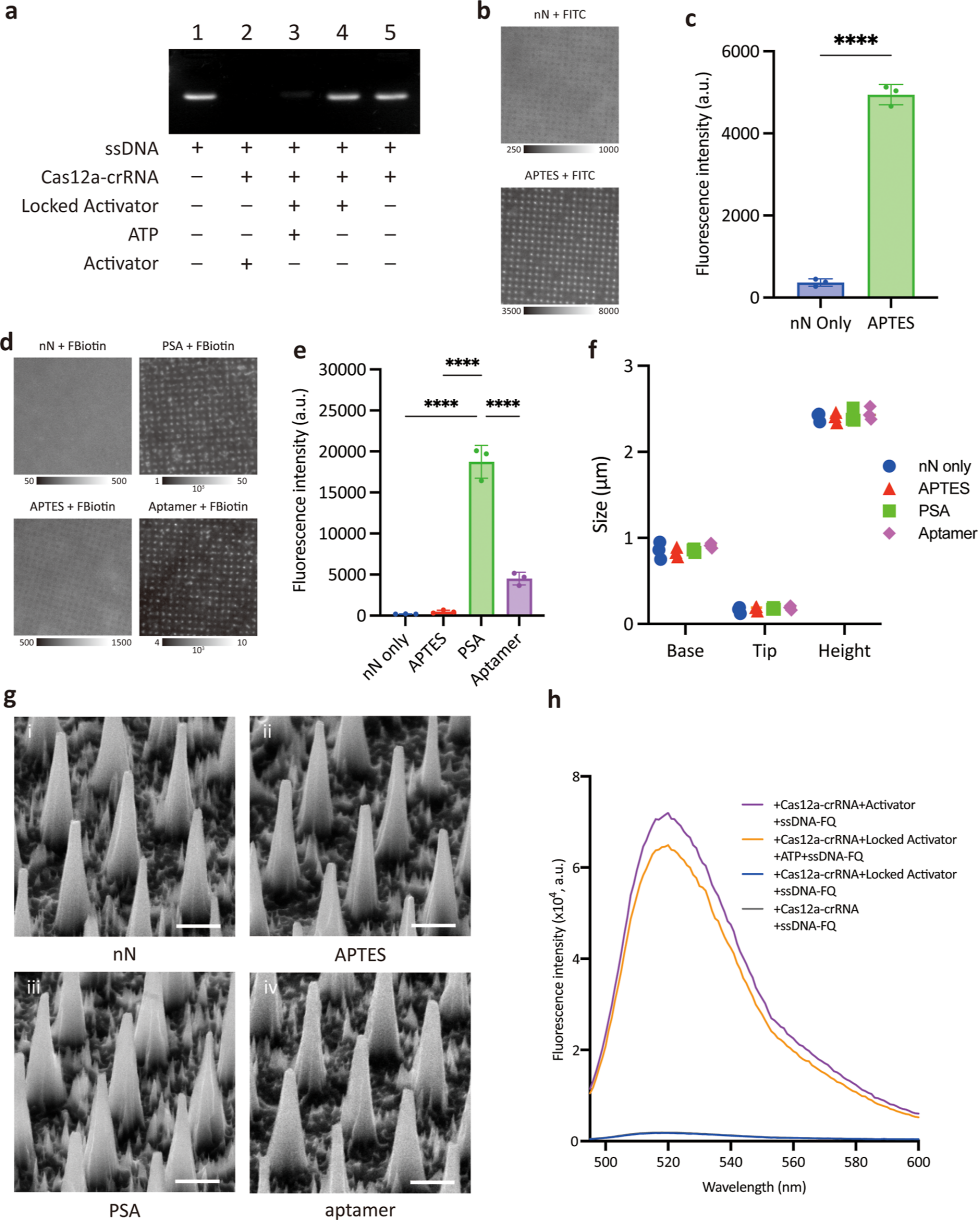
Validation of the nanoCRISPR sensor for ATP detection. (a) Agarose gel electrophoresis analysis of CRISPR/Cas12 activation upon ATP detection by the locked activator in solution. ssDNA without secondary structure was used as the substrate (lane 1: ssDNA, lane 2: ssDNA + Cas12a/crRNA + activator, lane 3: ssDNA + Cas12a/crRNA + locked activator + ATP, lane 4: ssDNA + Cas12a/crRNA + locked activator, and lane 5: ssDNA + Cas12a/crRNA). (b) Representative fluorescence microscopy images of bare nanoneedles (top) and APTES-functionalized nanoneedles following incubation with amine-reactive FITC. Scale bars 6 μm. Images are acquired at 16bit using the same conditions, but fluorescence is reported on different intensity scales, found under the image. (c) Quantification of fluorescence intensity from the microscopy images shown in (b) illustrating APTES conjugation on nanoneedles. Analysis is performed on five images from each of three independent nanoneedle chips. Data are reported as mean with standard deviation and each data point is reported in the graph. Statistical analysis is performed by unpaired *t*-test. ****p < 0.0001. (d) Representative fluorescence microscopy images of nanoneedles at different stages of functionalization following incubation with FITC-Biotin (FBiotin). Scale bars 6 μm. Images are acquired at 16bit using the same conditions, but fluorescence is reported on different intensity scales, found under the image. (e) Quantification of fluorescence intensity from the microscopy images shown in (d) illustrating successful aptamer conjugation on nanoneedles. The decrease in fluorescence following aptamer conjugation is proportional to the activator occupancy of biotin binding sites. Analysis is performed on five images from each of three independent nanoneedle chips. Data are reported as mean with standard deviation and each data point is reported in the graph. Statistical analysis is performed by 1-way analysis of variance with posthoc Tukey test. ****p < 0.0001. (f,g) SEM assessment of the nanoneedle chip at key stages of the nanoCRISPR assembly process. (f) Quantification of nanoneedles dimensions, and (g) SEM images of nanoneedles. (h) Fluorescent signal generation from the assembled nanoCRISPR sensor upon ATP detection (purple spectrum: activator immobilized on nanoneedles + Cas12a/crRNA + ssDNA F-Q, gray spectrum: bare nanoneedles + Cas12a/crRNA + ssDNA F-Q, orange spectrum: Locked Activator immobilized on nanoneedles + ATP + Cas12a/crRNA + ssDNA F-Q, and blue spectrum: locked activator immobilized on nanoneedles + Cas12a/crRNA + ssDNA F-Q). The gray and blue spectrum overlap in the graph as in both instances no activation is observed.

To determine the feasibility of our approach, we first verified the activation of Cas12a by target ATP by agarose gel electrophoresis analysis using a 50 nt ssDNA as the substrate (Figure 2a). The ssDNA (lane 1) was cleaved by the Cas12a in the presence of the activator (lane 2) but not in the absence of activator (lane 5), indicating that the activator was responsible for triggering the ssDNA cleavage of Cas12a/crRNA. In the presence of ATP and a locked activator (lane 3), we observed ssDNA digestion similar to the unlocked activator (lane 2), indicating that target ATP binding event effectively unlocked the activator and thus activated the cleavage effect on ssDNA. On the other hand, in absence of ATP the activator remained locked by the aptamer, as no cleavage (lane 4) was observed.

**Figure 3:**
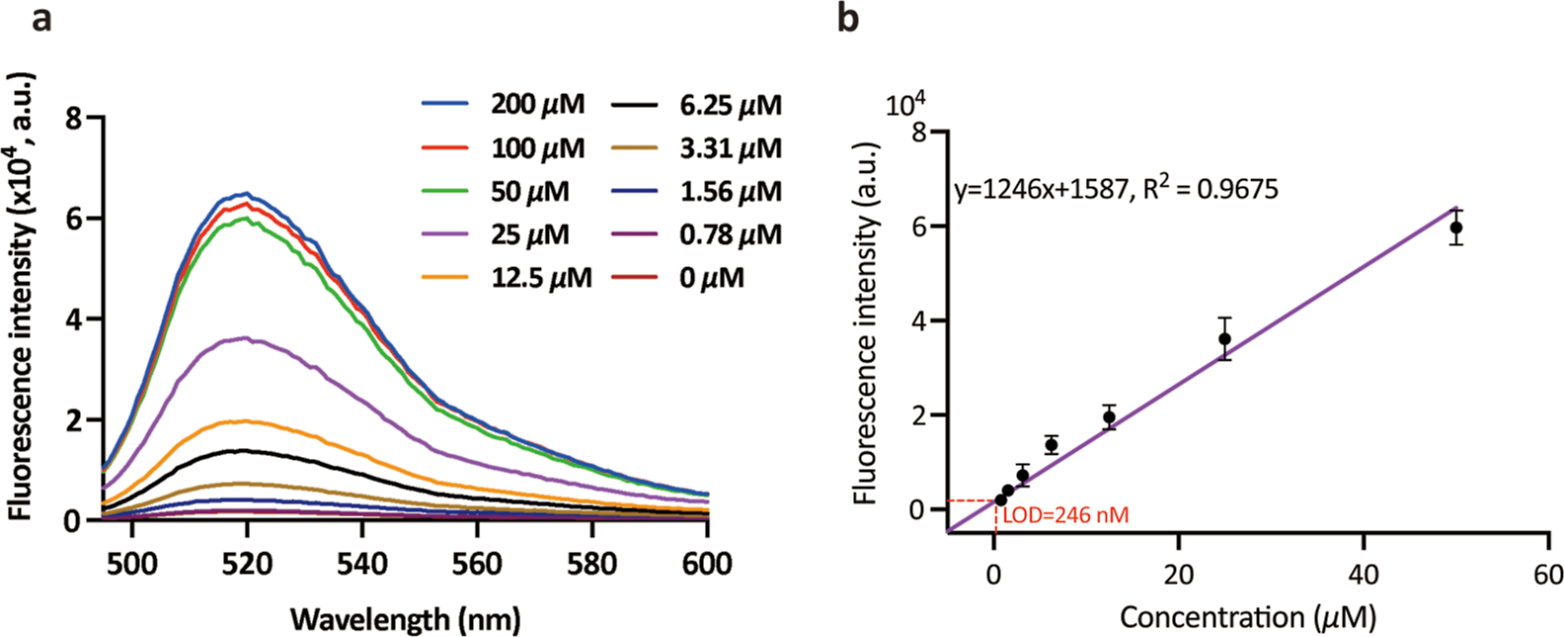
Performance of the nanoCRISPR sensor for the detection of ATP. (A) Fluorescence response of the nanoCRISPR in the presence of ATP ranging from 0 μM to 200 μM (0, 0.78, 1.56, 3.13, 6.25, 12.5, 25, 50, 100, and 200 μM) (b) linear regression of the fluorescence intensity at 520 nm as a function of ATP concentration. Data represent the average ± standard deviation from five measurements.

We then monitored the assembly of the sensing element on the nanoneedles. The nanoneedles were functionalized by using APTES to provide amine functional groups. The 13.5-fold increase in fluorescence following incubation with fluorescein isothiocyanate (FITC) compared to bare nanoneedles confirmed the presence and reactivity of the amine functional groups following APTES conjugation (Figure 2b,c). Polystrepatividin conjugation to the APTES-terminated nanoneedles was confirmed by the 97.5-fold increase in fluorescence following incubation with FITC-Biotin compared with bare nanoneedles (Figure 2d,e). The assembly of the aptamer sensing element was confirmed by the decrease in fluorescence to 24% of the value observed for PSA nanoneedles following FITC-Biotin incubation (Figure 2d,e). Such a decrease suggested that 76% of the available PSA binding sites on the surface were occupied by the aptamer sensing element. Scanning electron microscopy (SEM) revealed that nanoneedles remained intact across all steps of the sensor assembly, and retained their characteristic dimensions without accumulating any visible molecular adsorbate, thus preserving their functionality (Figure 3f,g). We then validated that the sensing element retained its functionality when assembled on nanoneedles (Figure 2h). When the activator strands were immobilized on nanoneedles, a remarkable fluorescence signal was obtained after incubation with ssDNA-FQ and Cas12a/crRNA (violet spectrum in Figure 2h). Instead, a negligible signal was observed when ssDNA-FQ and Cas12a/crRNA were incubated with nanoneedles in the absence of the activator (gray spectrum in Figure 2h). We then tested the locked activator immobilized on nanoneedles. We used the optimal molar ratio of two ATP aptamers per each activator; this ratio yielded minimal nonspecific unlocking of the activator as shown by the low background fluorescence when incubated with the Cas12a/crRNA + ssDNA-FQ in the absence of ATP (Figure S2).^13,17^ When this system was incubated with 200 μM ATP and Cas12a/crRNA with ssDNA-FQ, a strong fluorescence signal was observed (orange spectrum; Figure 2h). In contrast, when ATP was absent, a weak fluorescence signal was measured (dark blue spectrum; Figure 2h). Taken together, these results indicate that the sensor assembles effectively on the nanoneedles and can detect target ATP by activating the ssDNA cleavage activity of Cas12a, corroborating the results from agarose gel electrophoresis analysis.

### Sensitivity and Selectivity of nanoCRISPR

To examine the analytical performance of the proposed nanoCRISPR sensor, we measured the fluorescence spectra in the presence of ATP concentrations ranging from 0 to 200 μM. By evaluating the fluorescence intensity as a function of reaction time, we determined an optimal incubation of 20 min (Figure S3). The fluorescence signal gradually decreased with the decrease in concentrations of ATP (Figure 3). Furthermore, the signal was still visible even at the lowest assessed concentration of 0.78 μM, compared to the signal of a blank sample (Figure 3a). The fluorescence intensity measured at 520 nm linearly increased with ATP concentration in the range from 1.56 to 50 μM, with a linear fit *y* = 1246*x* + 1587 (Figure 3b). The *R*^2^ value of 0.97 indicated a robust linear relationship between fluorescence signal and ATP concentration. Using the LOD formula LOD = 3·*sb*/*m*, where *sb* is the standard deviation of fluorescence response for blank samples and *m* is the slope of the calibration curve, the LOD of nanoCRISPR for target ATP was estimated to be 246 nM. This LOD is approximately 5-fold lower than that of commercial ATP kits and comparable to that of other CRISPR-based sensing systems reported previously.^14–17^

**Figure 4:**
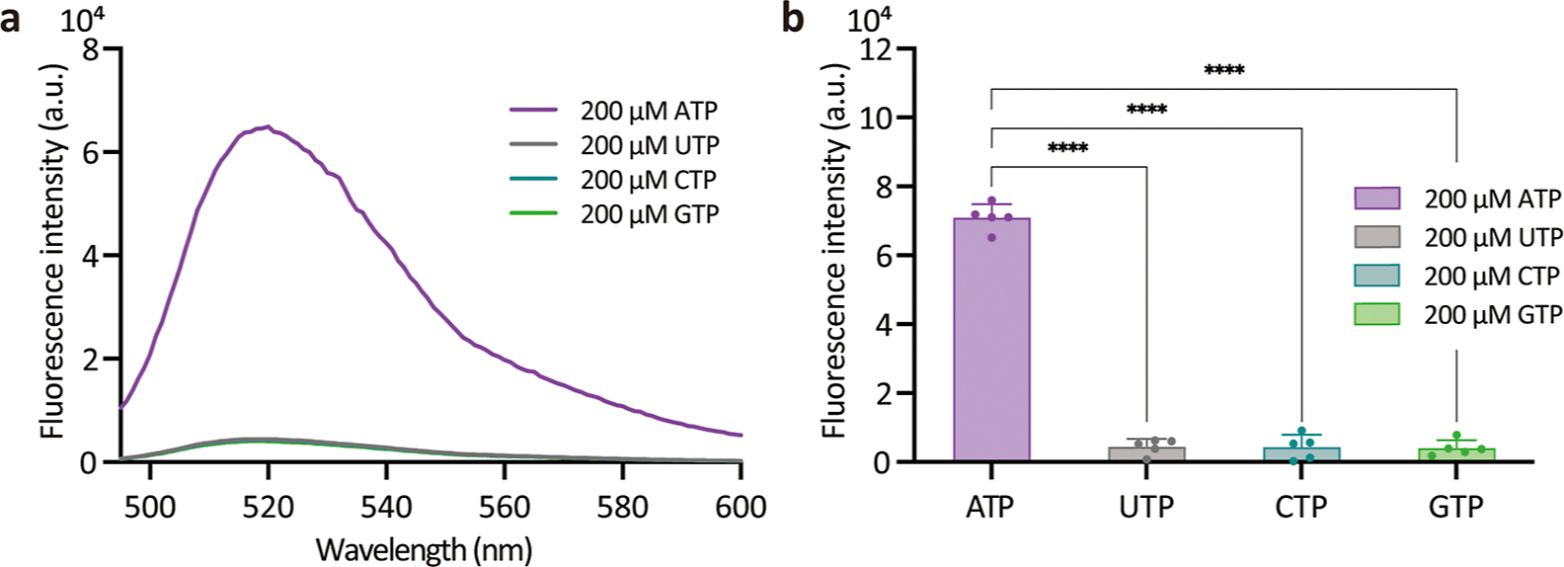
Selectivity of nanoCRISPR for ATP detection. (a) Fluorescence spectra recorded from nanoCRISPR after exposure to 200 μM ATP, UTP, CTP, and GTP. (b) Corresponding histograms for the fluorescence intensity at 520 nm as measured from the fluorescence spectra. Data represent mean plus standard deviation from five measurements. Individual measurements are reported in the graph. One way ANOVA with Tukey post-test; ****p < 0.0001.

The selectivity of nanoCRISPR was investigated by comparing the fluorescence response for ATP with that for the competing nucleotide-triphosphate analogues uridine 5′-triphosphate (UTP), cytidine 5′-triphosphate (CTP), and guanosine 5′-triphosphate (GTP) (Figure 4a) at 200 μM concentration. Fluorescence intensity at 520 nm for ATP was more than 14 times stronger compared to the analogues (Figure 4b). These results indicate that nanoCRISPR is sensitive to and highly specific for ATP.

### Intracellular Detection of ATP

**Figure 5:**
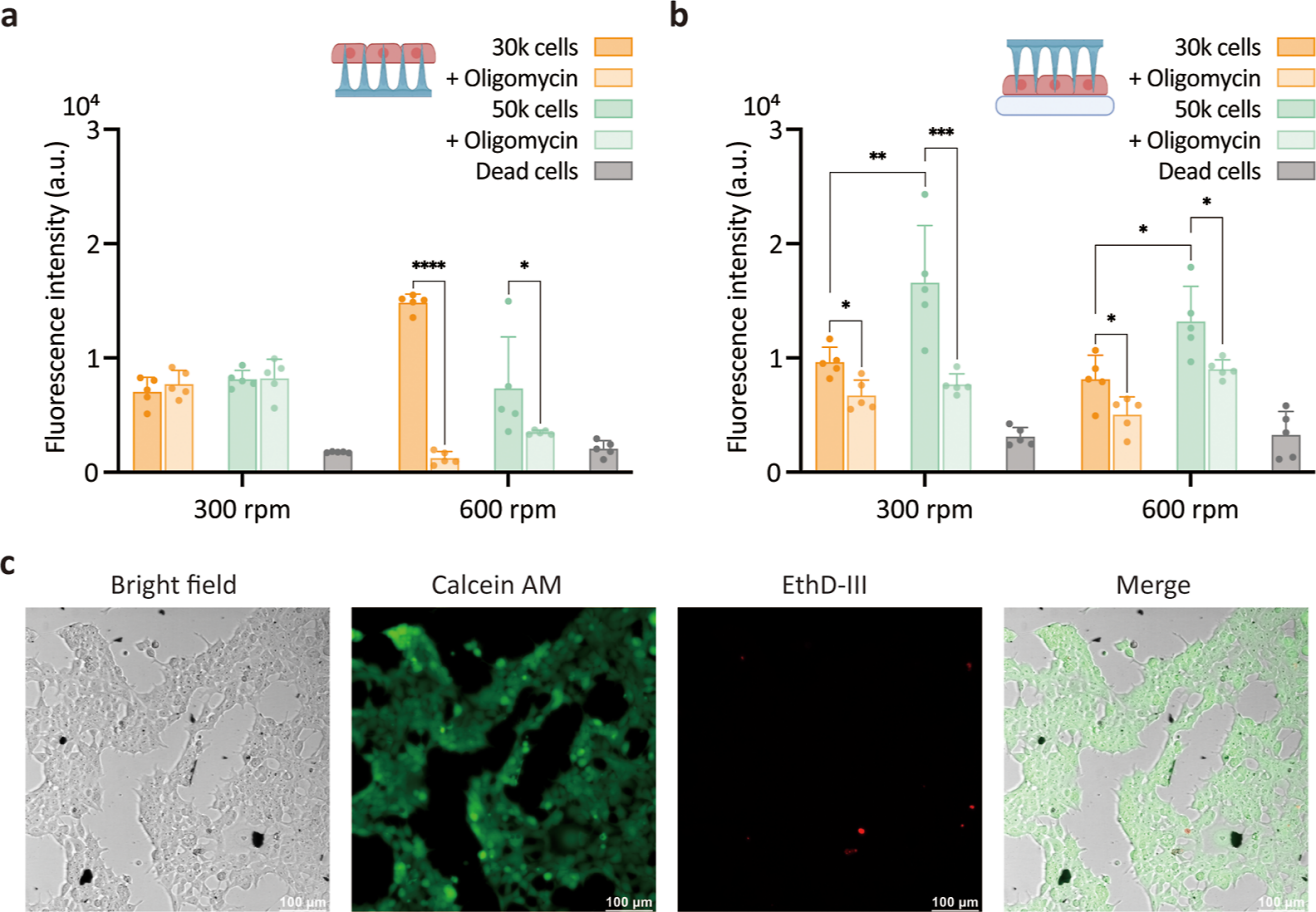
Intracellular detection of ATP using nanoCRISPR in live cells. (a) Comparison of the fluorescence signal detected at 520 nm for nanoCRISPR interfaced nanoneedles on the bottom. Individual groups include HEK-293 cells at 30k and 50k cell concentration, with and without oligomycin treatment and dead cells, at the two centrifugation speeds of 300 and 600 rpm. (b) Comparison of the fluorescence signal detected at 520 nm for nanoCRISPR interfaced nanoneedles on top. Individual groups include HEK-293 cells at 30k and 50k cell concentration, with and without oligomycin treatment and dead cells, at the two centrifugation speeds of 300 and 600 rpm. Data represent mean plus standard deviation from five measurements. Statistical analysis was performed by two-way analysis of variance with the Holm-Sidak multiple-comparison test. *p < 0.0332, **p < 0.021, ***p < 0.0002, and ****p < 0.0001. (c) LIVE/DEAD assay on 50k HEK-293 cells following nN-T interfacing with nanoCRISPR at 300 rpm. Left to right: bright field; green fluorescence from Calcein AM indicating live cells; red fluorescence from EthD-III indicating dead cells; merged channels. Scale bar is 100 μm.

To demonstrate the feasibility of our proposed nanoCRISPR approach for intracellular ATP sensing, we prepared cultures with 30,000 and 50,000 HEK-293 cells, and cells treated with oligomycin, which is a known inhibitor of mitochondrial ATP synthase. We evaluated the intracellular ATP detection performance based on the modality of interfacing by comparing cells cultured on nanoneedles (nanoneedles on bottom, nN-B) with nanoneedles placed over cells in culture (nanoneedles on top, nN-T) (Figure 5). All modalities of interfacing were assisted by centrifugation. For nN-B interfacing, a distinct fluorescence signal was obtained from all samples compared to that of dead cells (Figure 5a). However, the fluorescence intensities obtained from HEK-293 and oligomycin-treated HEK-293 cells were quite similar at a centrifugation speed of 300 rpm. A clear difference in fluorescence intensities between HEK-293 and oligomycin-treated HEK-293 was observed only when a centrifugation speed of 600 rpm was applied. Furthermore, the nN-B interfacing showed limited ability to discriminate between 30,000 and 50,000 cells in culture, regardless of centrifugation speed. On the other hand, for nN-T interfacing the difference between the fluorescence signal of untreated and oligomycin-treated cell was clearly seen at both 300 and 600 rpm (Figure 5b). The average relative signal difference between treated and untreated cells was ∼57%, which is ∼1.7 times larger than the signal difference measured for nN-B interfacing. Similarly, nN-T interfacing could discriminate between 30,000 and 50,000 cells at both centrifugation speeds. These results show that the nanoCRISPR sensor can detect intracellular ATP concentration and has better sensitivity when using nN-T interfacing.

Within the complex intracellular environment, nonspecific interactions such as enzymatic degradation or cross-target recognition can occur, reducing the sensitivity and specificity of the sensor. However, the markedly different fluorescence observed for oligomycin-treated cells and the minimal fluorescence arising from dead cells are indicators that our sensor retains sufficient intracellular specificity toward ATP.

Analogously to most ATP assays, our approach directly provides a relative assessment of ATP concentration. Absolute ATP quantification would require generating an intracellular calibration curve. However, the intracellular aptamer-based recognition occurs in a complex biological environment, where nonspecific interactions between the surface-bound biosensor and the environment, such as the formation of a biomolecular corona, can influence the recognition process. Thus, to appreciate the magnitude of the impact of such nonspecific interactions on the performance of our sensor, we estimated the single-cell ATP concentration using the linear fit of fluorescence intensity as a function of ATP concentration calculated in the absence of cells (Figure 3b) alongside an estimation of the volume and number of cells
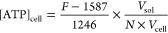


Where [ATP]_cell_ represents ATP cell concentration, *F* is the fluorescent signal at 520 nm, *V*_sol_ is the volume of the sensing solution, *V*_cell_ is the volume of an individual cell estimated at 16pL from measuring cells in suspension, and *N* is the number of cells interfaced. For nN-T interfacing, [ATP]_cell_ measured in untreated HEK-293 cells was 1.138 ± 0.26 mM (600 rpm, nN-T, 50k cells). This result is in agreement with previous reports indicating cellular ATP concentrations ranging from 1 to 10 mM and suggest that the complex intracellular environment does not significantly impair the functionality of our sensor.^60^

We determined the impact of nanoneedle interfacing on cells by the live/dead assay to assess the cell viability (Figure 5c). For nN-T interfacing, regardless of centrifugation speed, we could not detect evidence of dead cells, indicating that nanoCRISPR enables intracellular ATP detection without major cell perturbation.

## Conclusions

We developed nanoCRISPR as an intracellular ATP sensing platform, combining nanoneedle technology for intracellular access with aptamer ATP recognition and CRISPR/Cas12a amplification. We assembled on nanoneedles a locked-activator sensor capable of selectively recognizing ATP, resulting in unlocking of the Cas12a activator and triggering the cleavage of ssDNA-FQ by Cas12a, yielding a detectable fluorescent signal. NanoCRISPR can quantitatively detect ATP with an LOD of 246 nM and high selectivity against other triphosphate nucleotides. Moreover, by nanoneedle on top interfacing with the assistance of centrifugation, nanoCRISPR can detect intracellular ATP in living cells in 30 min. The sensor can discriminate between different intracellular ATP concentrations and different cell numbers without impacting cell viability. Future studies should systematically evaluate the variations in ATP detection performance due to nonspecific interactions and cross-reactivity within the complex intracellular environment. This approach opens the way to noninvasive longitudinal monitoring of cell viability for long-term culture and monitoring of the effects of challenges to cells. It is anticipated that the simple, rapid, and sensitive nanoCRISPR sensor can be applied in biomedical studies and cancer research for monitoring dynamic ATP levels and extended for detecting a range of other analytes in living sample.

## Experimental Section

### Materials

The all DNA and RNA sequences synthesized and purified with HPLC by Integrated DNA Technologies (UK) are listed in Table S1. Cas12a and related buffers were purchased from Integrated DNA Technologies. Adenosine 5′-triphosphate (ATP), CTP, GTP, UTP, and RNase inhibitor were purchased from NEW ENGLAND BioLabs Inc. (Ipswich, UK). NHS-Biotin was purchased from Sigma-Aldrich, Inc. (H1759–100 MG). Polystreptavidin R buffer was purchased from BioTeZ Berlin-Buch GmbH. 4-(2-hydroxyethyl)-1-piperazineethanesulfonic acid (HEPES), with APTES, and other chemicals were purchased from Sigma-Aldrich, Inc.

### Fabrication of Porous Silicon Nanoneedles

Porous silicon nanoneedles were fabricated according to established protocols.^31^ First, a 160 nm layer of silicon-rich silicon nitride was deposited by chemical vapor deposition onto p-type, 100 mm, ⟨100⟩ silicon wafers. Second, the substrate was patterned with a square array of dots having diameter of 600 nm and pitch of 2 μm, by UV photolithography. NR9–250P photoresist (Futurrex Inc., USA) was spin coated on the substrate to form a 220 nm thick layer, by using the following steps 500 rpm/1000 rpm/5 s, 4000 rpm/5000 rpm/40 s. Prebaking of the substrate was performed on a hot plate at 70 °C for 180 s; then, the substrate was placed in contact with the mask by hard vacuum contact by using a MA6 mask aligner (K. Suss GMBH, Germany) and exposed to UV. After exposure, postbaking of the substrate was performed on a hot plate at 100 °C for 60 s. The photoresist was developed in a dilution of the developer 3:1 RD6:H_2_O, for 12 s (Futurrex Inc., USA) and then rinsed with excess water and dried with a stream of N_2_. After photolithography, the pattern was transferred to the silicon nitride layer by reactive ion etching (RIE, NGP80 Oxford Instruments, UK) at 50 sccm CHF_3_, 5 sccm O_2_, 150 W forward power, 55 mTorr pressure, for 150 s. The remaining photoresist was stripped by acetone, and the substrate was rinsed with isopropanol and dried with stream of N_2_. The nitride pattern was then used to mask the silicon during metal assisted chemical etching (MACE), to form an ordered array of porous silicon pillars. Before MACE, the native oxide layer was removed by substrate dip in 10% HF (Honeywell, USA) for 2 min. This was immediately followed by electroless Ag deposition in 100 mL of 20 mM AgNO_3_ (Sigma-Aldrich), 10% HF for 2 min. The substrate was rinsed with water, then isopropanol, and dried with stream of N_2_. The substrate underwent MACE in 400 mL of a solution of 30 vol H_2_O_2_ (Sigma-Aldrich)/10% HF in volume ratio 1:99, for 3 min. To stop the etch, the wafer was dipped in H_2_O, rinsed with excess H_2_O and isopropanol, and dried with a stream of N_2_. The residual Ag was removed by dipping the substrate in gold etchant solution (Aldrich) for 10 min. The substrate was rinsed with excess H_2_O and isopropanol and dried with a stream of N_2_. The conical structure of the nanoneedles was obtained by RIE (NGP80, Oxford Instruments, UK) at 20 sccm SF_6_, 200 W forward power, 100 mTorr pressure, for 90 s. After the fabrication of the nanoneedles, the 100 mm wafer was diced in 4 × 4 mm square chips by using a dicing saw (Disco, DAD3220).

### Surface Functionalization of Nanoneedles

The fabricated nanoneedles were oxidized by O_2_ plasma for 10 min in the Low-pressure Plasma System (ZEPTO-W6, Diener electronic GmbH & Co). The oxidized surfaces of nanoneedle were functionalized with amine group by incubating with 2% APTES in ethanol for 3 h. The nanoneedles were then washed three times with ethanol and dried for 1 h at 100 °C. Next, NHS-Biotin (100 μg/mL) was coated to the nanoneedles for 1 h. After washing with PBS five times, the biotinylated nanoneedles were reacted with poly streptavidin (50 μg/mL) for 18 h and washed with DI water five times. Finally, the biotinylated locked activator probes (5 μM) were added to the polystreptavidin/biotin/nanoneedles array. After washing with PBS three times, ATP aptamer-1 and aptamer-2 (10 μM) in a hybridization buffer solution(5X SSC, 750 mM NaCl and 75 mM trisodium citrate) were added to the locked activator functionalized nanoneedles and incubated for 6 h, followed by washing twice with the rinsing buffer solution (2X SSC and 0.05 wt % Tween-20).

### Fluorescence Microscopy Characterization of nanoCRISPR Assembly

At every step of the assembly analyzed, three samples for each group were hard-mounted onto coverslips overnight (P36930, ProLong Gold Antifade Mountant). Five high-magnification images of each sample, including the fluorescent channel (FITC) and reflectance channel, were acquired by a Leica DMi8 microscope with a 63X 1.2 NA water objective. A custom MATLAB (R2021a) script was generated to measure the fluorescence intensity of individual nanoneedles. Briefly, the digital image processing script performed local edge detection, particle size exclusion, and segmentation to generate the nanoneedle mask based on the reflectance channel image. Finally, the mask was applied to extract the grayscale value of every single nanoneedle from the fluorescent channel (FITC), and the result was averaged across the whole image for analysis.

### SEM Characterization of NanoCRISPR Assembly

At every step of the assembly analyzed samples were washed in DI water and dehydrated in graded ethanol. SEM images were captured using a Carl Zeiss XB1540 Crossbeam SEM/FIB at tilted 45°. The tip width, height, and bottom width are measured manually using Fiji.

### Preparation of Cas12a/crRNA Complex and Detection of ATP

The 10 μM crRNA (10 μL) was preincubated with 62 μM dCas9 (1.6 μL) in PBS buffer at RT for 30 min. After the Cas12a/crRNA complex was created (1 μM), the complex was stored at 4 °C for up to 24 h before use. For the sensitivity analysis, 20 μL of reaction buffer (40 mM HEPES, 100 mM NaCl, 20 mM MgCl_2_) containing different concentrations of ATP was added onto the locked activator functionalized nanoneedle. After incubation of 5 min, 40 μL of 100 nM Cas12a/crRNA complex, 30 μL of 100 nM ssDNA F-Q, and 10 μL of reaction buffer were added and incubated for 20 min at 25 °C. After that, the enzymatic reaction was stopped by heating at 60 °C for 5 min, and the fluorescence data was collected. For selectivity analysis, different nucleoside triphosphates were added into the locked activator functionalized nanoneedle, and other procedures were the same as ATP sensitivity analysis.

### Cell Culture

HEK293-T cells were cultured in DMEM medium (Gibco) supplemented with 10% fetal bovine serum (FBS, Gibco) and 1% penicillin–streptomycin (Gibco) and were incubated at 37 °C with 5% CO_2_. Mycoplasma contamination of the cells was tested to their standard levels of stringency. Cells were washed three times with DPBS (Sigma-Aldrich) and trypsinized. Cell were resuspended in DMEM and counted using hemocytometer before being seeded into the 96-well plate for the experiment. Oligomycin (Selleck Chemicals) was prepared 1 mg/mL as stock solution according to the manufacturer’s protocol, and 2 μL was applied to the 200 μL cell culture system to make a final working concentration of 10 μg/mL for treatment.

### Intracellular Detection of ATP

For nanoneedles on top (nN-T) interfacing, the nanoCRISPR device was placed to float on a culture medium with the nanoneedles facing toward cells, and then, the whole setup was centrifuged at various speeds for 5 min. After centrifugation, more medium was immediately added to the culture well to remove the nanoneedle sensor by lifting the cells. For nanoneedles on bottom interfacing (nN-B), the nanoneedles were placed at the bottom of a 96-well plate, and 200 μL of medium containing the desired amount of cells was added. The whole setup was centrifuged at various speeds for 5 min. After removal, for either interfacing methodology, the nanoneedle was washed three times with PBS. Next, 100 nM Cas12a/crRNA complex and 100 nM ssDNA F-Q were added and incubated for 35 min at 25 °C. After that, the enzymatic reaction was stopped by heating at 60 °C for 5 min and the fluorescence data were collected.

### Live/Dead Assay

Calcein AM (BD Pharmingen) 5 mM stock solution in DMSO was prepared and stored at −20 °C according to the protocol. Live/dead staining mix was prepared by adding Calcein AM (1:2000) and Ethidium Homodimer III (EthD-III, Biotium) (1:1000) to Hank’s Balanced Salt Solution (HBSS, Gibco). Cells in the 96-well plate were HBSS washed once before adding the live/dead staining mix. After incubation at room temperature in the dark for 30 min, the cells were washed with HBSS once and then supplemented and left in HBSS for microscope imaging.

### Instruments

Fluorescence data were collected in a CLARIOstarPlus plate reader using 485 nm excitation and 520 nm emission. Gel imaging was carried out using U:Genius^3^ system. Images were acquired by a DMi8 inverted microscope with a 20X 0.8 NA dry objective.

## Supporting Information Available

Sequences of oligonucleotides used in this study; schematic representation of surface functionalization of nanoneedles; evaluation of the aptamers locking effect; and fluorescence response of the nanoCRISPR as a function of reaction times (PDF)
