# Supplementary material for: CRISPR/Cas-Assisted Nanoneedle Sensor for Adenosine Triphosphate Detection in Living Cells
Source: ACS Appl Mater Interfaces. 2023 Sep 28;15(43):49964–73. doi: 10.1021/acsami.3c07918 (PMC10623508; doi:10.1021/acsami.3c07918)
Supplement: Supplementary file 1 — am3c07918_si_001.pdf [file am3c07918_si_001.pdf]

CRISPR/Cas-assisted Nanoneedle Sensor for ATP detection in Living Cells

Hongki Kim[1,2], Chenlei Gu[1,3], Salman Ahmad Mustfa[1,†], Davide Alessandro Martella[1], Cong Wang[1,3], Yikai Wang[1,3], and Ciro Chiappini[1,3]*

1 Centre for Craniofacial and Regenerative Biology, King's College London, London, SE1 9RT, UK

2 Department of Chemistry, Kongju National University, Gongju 32588, Republic of Korea

3 London Centre for Nanotechnology, King's College London, London, SE1 9RT, UK

† current address: AstraZeneca, Granta Park, Great Abington, Cambridge, CB21 6GH, United Kingdom

Correspondence to: ciro.chiappini@kcl.ac.uk

Table S1. Oligonucleotides used in this study

	**Sequence (5′ → 3′)**
Cas12a guide crRNA	rUrArA rUrUrU rCrUrA rCrUrA rArGrU rGrUrA rGrArU rArArG rGrUrU rUrGrU rGrUrG rUrUrU rArCrC rUrG
Activator	CCC AGG TAA ACA CAC AAA CCT T
Aptamer-1	ACC TGG GGG AGT ATT GCG GAG GAA GGT TTG TGT
Aptamer-2	GTT TAC CTG GGG GAG TAT TGC GGA GGA AGG T
Immobilized-Activator	/Biotin TEG/ATA TAT ATA TAT ATA TAT ATC CCA GGT AAA CAC ACA AAC CTT
ssDNA F-Q	/56-FAM/TT ATT /3IABkFQ/

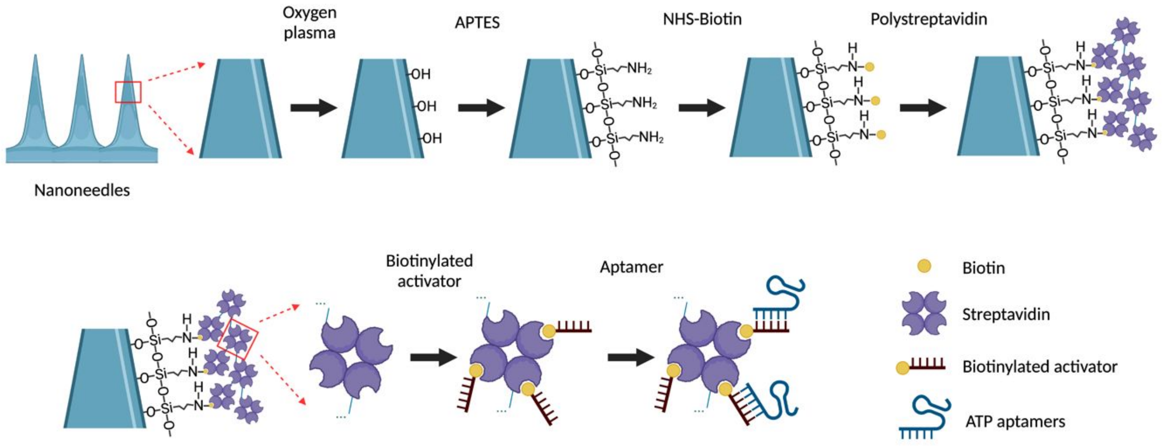

Figure S1. Schematic representation of surface functionalization of nanoneedles. The surface of nanoneedles is oxidised by Oxygen plasma. The oxidised surface is functionalized with amine group by incubating with APTES. NHS-Biotin is conjugated to the amine-functionalized nanoneedles and the biotinylated nanoneedles are reacted with poly-streptavidin. The biotinylated activator is subsequently immobilized onto nanoneedles via biotin–avidin interaction. Lastly, ATP aptamers are employed to lock the activator on the nanoneedles.

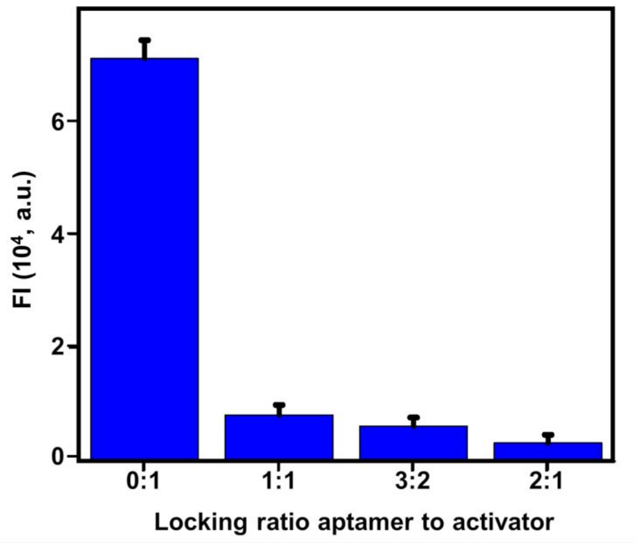

Figure S2. Evaluation of the aptamers locking effect. The fluorescent signals were obtained by immobilizing various ratios of activator and aptamer on the nanoneedles. Data represent mean plus standard deviation from five measurements.

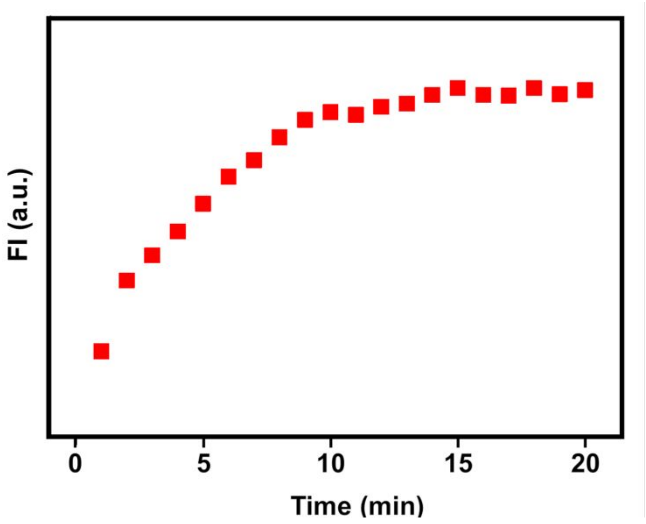

Figure S3. Fluorescence response of the nanoCRISPR as a function of reaction times.
